# Kinetic modeling of tricarboxylic acid cycle and glyoxylate bypass in Mycobacterium tuberculosis, and its application to assessment of drug targets

**DOI:** 10.1186/1742-4682-3-27

**Published:** 2006-08-03

**Authors:** Vivek Kumar Singh, Indira Ghosh

**Affiliations:** 1 Bioinformatics Centre, University of Pune, Pune-411007, India

## Abstract

**Background:**

Targeting persistent tubercule bacilli has become an important challenge in the development of anti-tuberculous drugs. As the glyoxylate bypass is essential for persistent bacilli, interference with it holds the potential for designing new antibacterial drugs. We have developed kinetic models of the tricarboxylic acid cycle and glyoxylate bypass in *Escherichia coli *and *Mycobacterium tuberculosis*, and studied the effects of inhibition of various enzymes in the *M. tuberculosis *model.

**Results:**

We used *E. coli *to validate the pathway-modeling protocol and showed that changes in metabolic flux can be estimated from gene expression data. The *M. tuberculosis *model reproduced the observation that deletion of one of the two *iso*citrate lyase genes has little effect on bacterial growth in macrophages, but deletion of both genes leads to the elimination of the bacilli from the lungs. It also substantiated the inhibition of *iso*citrate lyases by 3-nitropropionate. On the basis of our simulation studies, we propose that: (i) fractional inactivation of both *iso*citrate dehydrogenase 1 and *iso*citrate dehydrogenase 2 is required for a flux through the glyoxylate bypass in persistent mycobacteria; and (ii) increasing the amount of active *iso*citrate dehydrogenases can stop the flux through the glyoxylate bypass, so the kinase that inactivates *iso*citrate dehydrogenase 1 and/or the proposed inactivator of *iso*citrate dehydrogenase 2 is a potential target for drugs against persistent mycobacteria. In addition, competitive inhibition of *iso*citrate lyases along with a reduction in the inactivation of *iso*citrate dehydrogenases appears to be a feasible strategy for targeting persistent mycobacteria.

**Conclusion:**

We used kinetic modeling of biochemical pathways to assess various potential anti-tuberculous drug targets that interfere with the glyoxylate bypass flux, and indicated the type of inhibition needed to eliminate the pathogen. The advantage of such an approach to the assessment of drug targets is that it facilitates the study of systemic effect(s) of the modulation of the target enzyme(s) in the cellular environment.

## Background

Tuberculosis is an ancient disease that has plagued humans for centuries, and presently there is an urgent need for new drugs to combat drug-resistant tuberculosis and shorten the time of tuberculosis therapy. Tuberculosis treatment is lengthy because of a population of persistent bacilli that is not effectively eliminated by current drugs.

The persistent bacilli primarily use fatty acids as their carbon source [1]. This makes the glyoxylate bypass, consisting of *iso*citrate lyase (ICL) and malate synthase (MS), essential for the bacterium; in its absence there will be no net formation of the intermediates required for synthesizing cellular materials. Inhibition of both ICL1 (prokaryotic-like isoform) and ICL2 (eukaryotic-like isoform) has been shown to block the growth of *M. tuberculosis *in macrophages and in mice [2]. Hence, interference with the glyoxylate bypass is a potential approach to the design of new drugs against persistent mycobacteria. This is consistent with the suggestion that the regulation of *M. tuberculosis *metabolism in response to the environment of the bacterium makes large contributions to its virulence [3].

At the branch point of the tricarboxylic acid (TCA) cycle and glyoxylate bypass, *iso*citrate dehydrogenase (ICD), involved in the TCA cycle, and ICL, involved in the glyoxylate bypass, compete for the same substrate, namely *iso*citrate (ICIT). In *Escherichia coli*, flux at this branch point is predominantly controlled through the reversible inactivation of ICD by phosphorylation, catalyzed by ICD-kinase [4]. We have already identified the kinase in *M. tuberculosis*, equivalent to ICD-kinase in *E. coli*, that is responsible for reversible inactivation of ICD1 (Rv3339c) by phosphorylation [5]. Moreover, a method has been described for inhibiting a metabolic pathway that is essential for the viability of a microorganism by diverting the substrate to a different metabolic pathway, and it has been suggested that inhibiting ICD1-kinase could inhibit the flux through the glyoxylate bypass in *M. tuberculosis *[5]. Since inhibition of ICD1-kinase would increase the amount of dephosphorylated (active) ICD1, the flux through the glyoxylate bypass would be diminished. However, enzymes are not isolated entities in living organisms but act as components of systems, so the effect of modulation of any enzyme activity on a metabolic flux depends on the properties of the other enzymes in the pathway concerned [6].

Metabolic Control Analysis (MCA) is a theoretical framework that relates the systemic properties of a metabolic system to the properties of its components, in particular the enzymes, in a quantitative manner [6]. Application of MCA to the identification of potential drug targets is exemplified by glycolysis in *Trypanosoma brucei *[7-9]. MCA also gives insight into the cellular effect(s) of inhibition of a particular enzyme. Eisenthal *et al*. [9] suggested two basic metabolic methods for killing an organism: decreasing the flux through an essential metabolic pathway to a nonviable level, or increasing the concentration of a metabolite to a toxic level. Therefore, if inhibition of an enzyme kills an organism, MCA can elucidate the mechanism involved.

Since modulation of target enzyme(s) activity is usually aimed at altering the cell's metabolic profile, knowledge of the metabolic profile is important for identifying the target. Recent experiments have shown a positive correlation between mRNA levels measured by DNA microarrays and protein abundance in both *E. coli *[10] and yeast cells [11,12], so the gene expression profile could be connected to the metabolic profile via simulation of the pathway under study. In *E. coli*, the *in vivo *kinetic parameters required for estimating the metabolic profile of most enzymes are available when the organism is grown using glucose as the carbon source [13]. In contrast, when acetate is used as the carbon source, the gene expression profile of the TCA cycle and glyoxylate bypass enzymes differed from that found with glucose [14]. The corresponding metabolic flux distributions in central metabolic pathways under both growth conditions are known [15], so this seems an ideal system for testing the hypothesis that the gene expression profile can be connected with the metabolic profile via simulation of the pathway under study.

In this communication, we describe the construction of a kinetic model of the TCA cycle and glyoxylate bypass in *M. tuberculosis*, and we study the likely metabolic consequences of inhibiting ICLs and ICD1-kinase. To the best of our knowledge, this is the first attempt to model any specific metabolic pathway in *M. tuberculosis*, and no kinetic model is available for the TCA cycle and glyoxylate bypass in this bacterium. Initially, we constructed a kinetic model for the TCA cycle and glyoxylate bypass in *E. coli *to validate the pathway modeling protocol used and to test how well the metabolic profile correlates with the gene expression profile while trying to predict the metabolic flux distribution using the gene expression data.

**Figure 1:**
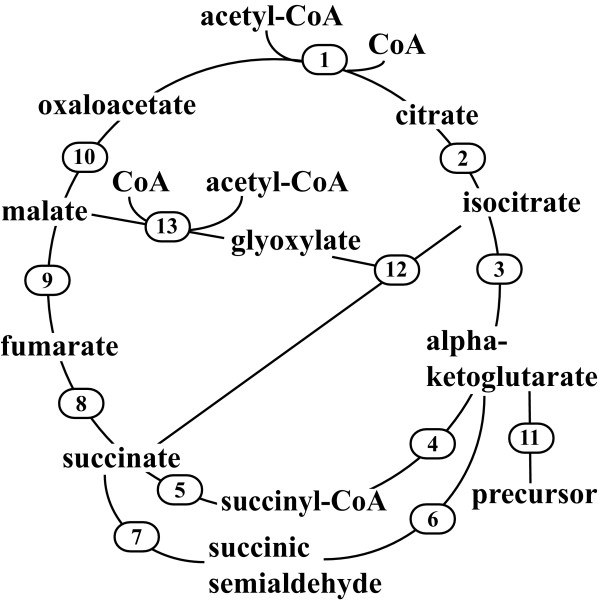
**TCA cycle and glyoxylate bypass reactions considered in *E. coli *and *M. tuberculosis *models**. Reactions 1, 2, 3, 5, 8, 9, 10, 11, 12 and 13 were present in all the models; reaction 4 was present only in the *E. coli *model and *M. tuberculosis *model-1, but absent from *M. tuberculosis *model-2; and reactions 6 and 7 were present in the *M. tuberculosis *models, but absent from *E. coli *model. 1, CS; 2, ACN; 3, ICD in *E. coli *model and ICD1 and ICD2 in *M. tuberculosis *models; 4, KDH; 5, ScAS; 6, KGD; 7, SSADH; 8, SDH; 9, FUM; 10, MDH; 11, fraction of αKG utilized for precursor biosynthesis (SYN); 12, ICL in *E. coli *model and ICL1 and ICL2 in *M. tuberculosis *models; 13, MS.

The biochemical reactions considered for the models are shown in figure 1 and the metabolites with known concentrations are listed in table 1. In *M. tuberculosis *H37Rv strain there are two isoforms of ICD [17], ICD1 (Rv3339c) and ICD2 (Rv0066c), and two isoforms of ICL [17,18], ICL1 (Rv0467) and ICL2 (Rv1915 and Rv1916). In addition, the inability of Nathan and co-workers to detect α-ketoglutarate dehydrogenase (KDH) activity in *M. tuberculosis *[13] was taken into account while constructing the model. *M. tuberculosis *model-1 represents a standard TCA cycle and glyoxylate bypass with KDH present, while model-2 lacks KDH activity. Our aim was to check the metabolic consequences of the presence and absence of KDH in this organism.

**Table 1:** Metabolites of the models with known concentrations (with references indicated in square brackets)

***Escherichia coli***			***Mycobacterium tuberculosis***	
**Metabolite**	**Concentration in glucose condition (in mM)**	**Concentration in acetate condition (in mM)**	**Metabolite**	**Concentration (in mM)**

acetyl-CoA	0.5 [16]	0.5 [16]	succinate	2.464 (derived from Tian et. al [13])
citrate	3 [16]	9 [16]	fumarate	0.08528 (derived from Tian et. al [13])
*iso*citrate	0.018^a ^[16]	0.15 [16]	malate	0.408 (derived from Tian et. al [13])
succinate	0.6 [16]	6 [16]	oxaloacetate	0.0003 (assumed)
malate	1.8 [16]	5 [16]	CoA	0.0001 (assumed)
oxaloacetate	0.004^b^	0.0014 (assumed)		
CoA	0.0001 (assumed)	0.0001 (assumed)		

^a^Isocitrate concentration was inferred from a graph shown by Walsh et. al [16]. The value in the graph was 0.025 mM at 30 minutes after addition of glucose to the medium, but it had a negative slope, so, a value of 0.018 mM was taken.

^b^Taken as 2.4 times the concentration of oxaloacetate under growth on acetate because flux leading to the synthesis of oxaloacetate under growth on glucose is 2.4 times of that under growth on acetate [15].

## Results and discussion

### Steady state solution for the models

Steady state fluxes in the *E. coli *model (table 2) were compared to the experimental fluxes given by Zhao *et al*. [15]; the net fluxes were expressed in relative units. The unit conversion is described in methods section. The steady state fluxes calculated from the model accorded with the experimental fluxes [15] (table 3), thus validating the protocol used.

**Table 2:** Steady state fluxes computed for *E. coli *model.

**Reaction step**	**Growth on glucose (mM/min)**	**Growth on acetate (mM/min)**
CS	4.187	8.006
ACN	4.187	8.006
ICD	4.179	6.125
KDH	3.394	5.916
ScAS	3.394	5.916
SDH	3.401	7.798
FUM	3.401	7.798
MDH	3.409	9.679
SYN	0.786	0.209
ICL	0.008	1.882
MS	0.008	1.882

**Table 3:** Comparison of the experimental fluxes to that computed from *E. coli *model. The reaction step SYN was not explicitly mentioned by Zhao *et al*. [15], but was shown by a branch from αKG.

**Reaction step**	**Growth on glucose (Experimental)**	**Growth on glucose (Simulation)**	**Growth on acetate (Experimental)**	**Growth on acetate (Simulation)**
CS	50	50	73.4	73.4
ACN	50	50	73.4	73.4
ICD	50	49.9	52.8	56.1
KDH	40.6	40.5	51.0	54.2
ScAS	40.6	40.5	51.0	54.2
SDH	40.6	40.6	71.6	71.5
FUM	40.6	40.6	71.6	71.5
MDH	40.6	40.7	86.3	88.7
SYN	9.4	9.4	1.8	1.9
ICL	0	0.1	20.6	17.2
MS	0	0.1	20.6	17.2

Since the maximal reaction rates (Vmax) of the enzymes during growth on acetate were estimated using gene expression data, it is possible to estimate the changes in metabolic flux distribution due to changes in gene expression via simulation of the biochemical pathway under study. This was also noted in the study of branched chain amino acid biosynthesis in *E. coli *[19].

The steady state fluxes in the *M. tuberculosis *model-1 (standard TCA cycle) and model-2 (absence of KDH activity) are shown in table 4. The fluxes in the two models of the *M. tuberculosis *TCA cycle and glyoxylate bypass are similar, with the following exceptions. (i) The entire flux from α-ketoglutarate (αKG) towards the TCA cycle passes through the α-ketoglutarate decarboxylase (KGD) and succinic semialdehyde dehydrogenase (SSADH) steps in model-2 (which has no other branch from αKG that continues in TCA cycle); in model-1, about 84% of the flux from αKG passes through KDH and the remaining 16% through KGD and SSADH, but the total flux from αKG continuing in the TCA cycle is almost the same in both models. (ii) Flux was observed through the succinyl-CoA synthetase (ScAS) step in model-1 but was negligible in model-2. This is expected because KDH converts αKG to succinyl-CoA, and succinyl-CoA must be converted to succinate (SUC) for the continuation of the TCA cycle. This conversion is brought about by ScAS. Model-2 does not require ScAS because it converts αKG directly to SUC using KGD and SSADH. The steady state fluxes computed from the two models showed minor differences, but the turnover of the TCA cycle and glyoxylate bypass was similar in both models, indicating that *M. tuberculosis *can manage without a functional KDH. Thus, this study illustrates that at the metabolic level, the absence of KDH activity has no effect on the net flux through the TCA cycle and glyoxylate bypass.

**Table 4:** Steady state fluxes computed for *M. tuberculosis *model-1 and model-2 (in persistent mycobacteria)

**Reaction step**	**Fluxes in model-1 (mM/min)**	**Fluxes in model-2 (mM/min)**
CS	0.988	0.988
ACN	0.988	0.988
ICD1	0.653	0.650
ICD2	0.331	0.333
KDH	0.797	-
ScAS	0.797	-5.65892 × 10^-11^
KGD	0.154	0.950
SSADH	0.154	0.950
SDH	0.955	0.955
FUM	0.955	0.955
MDH	0.959	0.959
SYN	0.034	0.034
ICL1	0.004	0.004
ICL2	0.000	0.001
MS	0.004	0.005

On the basis of the finding of Tian *et al*. [13], i.e. that KDH activity is absent in *M. tuberculosis*, and of the observation that there is little difference between the two models in the turnover of the TCA cycle and glyoxylate bypass, *M. tuberculosis *model-2 was taken as the reference model in the remaining parts of this study.

### Inactivation of ICDs in *M. tuberculosis *model

**Figure 2:**
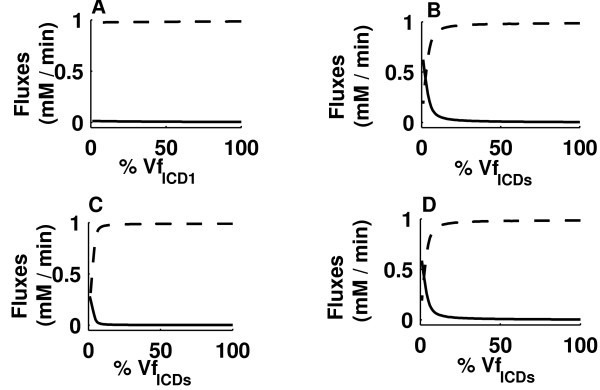
**Effect on the flux through ICDs and ICLs with varying Vf_ICD1 _and Vf_ICD2_**. Effects of varying (A) Vf_ICD1 _alone, (B) both Vf_ICD1 _and Vf_ICD2 _simultaneously (abbreviated as Vf_ICDs_), (C) Vf_ICD1 _and Vf_ICD2 _simultaneously (abbreviated as Vf_ICDs_) with ICL1 reaction removed from the model to simulate deletion of gene encoding ICL1, (D) Vf_ICD1 _and Vf_ICD2 _simultaneously (abbreviated as Vf_ICDs_), with ICL2 reaction removed from the model to simulate deletion of gene encoding ICL2. Broken line represents the sum of flux through ICD1 and ICD2, and solid line represents the sum of flux through ICL1 and ICL2.

Inactivation of ICD1, which is brought about by ICD1-kinase, leads to a change in the number of active ICD1 molecules. Since Vmax is a function of the amount of enzyme, any change in the amount of enzyme will affect the Vmax. Therefore, varying Vmax for ICD1 from 1% to 100% was used to monitor the effect of inactivation of ICD1 by ICD1-kinase. Since there is no information about any such kinase for ICD2, the activity value was kept at 100%. Plots of the sum of flux through ICD1 and ICD2 (J_ICD1 _+ J_ICD2_) and the sum of flux through ICL1 and ICL2 (J_ICL1 _+ J_ICL2_) against Vmax for the forward ICD1 reaction (Vf_ICD1_) (figure 2A) showed that even at 99% inactivation there was no perceptible flux through the glyoxylate bypass. We then studied the effect of inactivation of ICD2 by a hypothetical inactivator, along with the inactivation of ICD1. The plot of J_ICD1 _+ J_ICD2 _and J_ICL1 _+ J_ICL2 _against Vmax for the ICD1 and ICD2 forward reactions (Vf_ICD1 _and Vf_ICD2 _respectively) (figure 2B) showed that the flux through the glyoxylate bypass (J_ICL1 _+ J_ICL2_) starts to increase after Vf_ICD1 _and Vf_ICD2 _have fallen to approximately 30% of the original values, and becomes equal to J_ICD1 _+ J_ICD2 _when Vf_ICD1 _and Vf_ICD2 _have fallen to about 3% of the original values. Thus, flux through the glyoxylate bypass was observed only when both ICD1 and ICD2 were more than 70% inactivated. Inactivation of ICD1 has already been demonstrated experimentally [5], but no such phosphorylation-induced inactivation of ICD2 has been reported. The possibility of inactivation of ICD2 along with ICD1 in persistent mycobacteria, leading to an up-regulation of flux through the glyoxylate bypass, is suggested by our study. A novel protein might bring about this inactivation, or the kinase that acts on ICD1 might also act on ICD2. Since no differential expression of ICD1 and ICD2 has been reported in the literature, both the ICDs were kept active in our study. Interestingly, the model also suggests that if 30% or more of ICD1 and ICD2 are in the active state, there will be no flux through the glyoxylate bypass. Since the glyoxylate bypass is essential for persistent bacilli, they would perish under such conditions. Inhibition of ICD1-kinase and/or the proposed inactivator of ICD2 would increase the amount of active ICD1 and/or ICD2 respectively, suggesting that this is a potential target for the development of drugs against persistent mycobacteria.

### Deletion of genes encoding ICLs in *M. tuberculosis *model

McKinney and co-workers showed that deletion of either of the genes *icl1 *or *icl2 *had little effect on mycobacterial growth in macrophages or in mice [2]. In our model, deletion of *icl1 *could be simulated by deleting the ICL1 reaction. Plots of J_ICD1 _+ J_ICD2 _and J_ICL2 _as a function of Vf_ICD1 _and Vf_ICD2 _(figure 2C) showed that more than 90% inactivation of both ICD1 and ICD2 is required to allow a perceptible flux through the glyoxylate bypass in the absence of ICL1. In contrast, when both ICLs were present, 70% inactivation of both ICD1 and ICD2 sufficed to allow a flux through the glyoxylate bypass (figure 2B). Simulating *icl2 *gene deletion showed only a marginal difference in the flux through the glyoxylate bypass or in J_ICD1 _+ J_ICD2 _when plotted against Vf_ICD1 _and Vf_ICD2 _(figure 2D), compared to the fluxes observed in the presence of both ICLs (figure 2B). Thus, the model correctly simulates the experimental observation that deletion of either of the two ICL genes has little effect on the growth of mycobacteria in macrophages and in mice [2]. It also shows that a flux of approximately 26% through the glyoxylate bypass remains in the absence of *icl1*, compared to the flux when both ICLs are present (with Vf_ICD1 _and Vf_ICD2 _kept at 5% of the original values). In the absence of *icl2*, the flux through the glyoxylate bypass decreases only by 7.6% compared to the flux in presence of both ICLs (with Vf_ICD1 _and Vf_ICD2 _kept at 5% of the original values). Such a reduction in flux due to the deletion of either of the two ICL genes would be too small to lead to elimination of the bacilli.

### Competitive inhibition of ICLs

**Figure 3:**
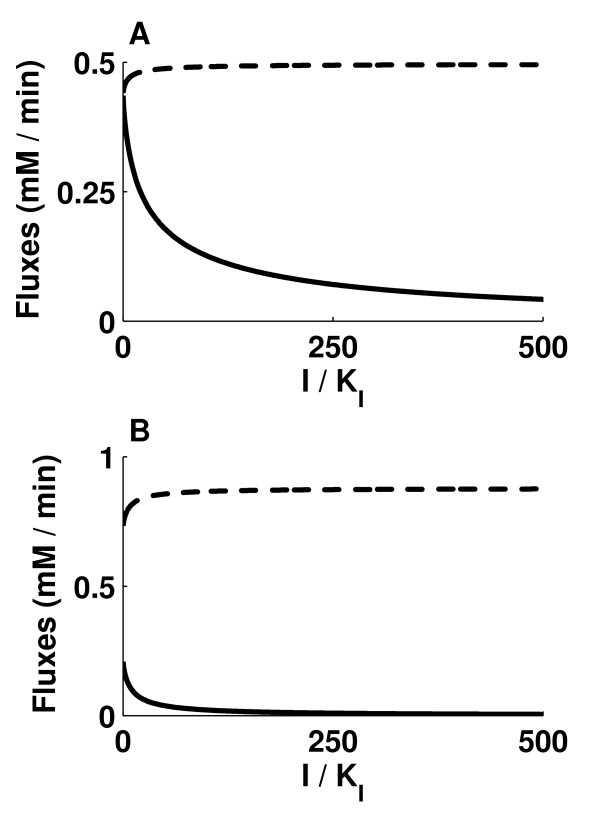
**Competitive inhibition of ICLs by an inhibitor with concentration I and inhibitor constant K_I_**. Inhibition of ICL1 and ICL2, with Vf_ICD1 _and Vf_ICD2 _both kept at (A) 2.5% of the original values, (B) 5% of the original values. Broken line represents the sum of flux through ICD1 and ICD2, and solid line represents the sum of flux through ICL1 and ICL2. The effect of inhibitor is shown by varying the ratio of I/K_I_.

The rate equations of the ICL1 and ICL2 reactions were modified to account for competitive inhibition, i.e. competition against *iso*citrate, as shown in equation (1). The ratio of inhibitor concentration to inhibitor constant (I/K_I_) was assumed to be the same for both ICL1 and ICL2. Two simulations were performed, one with Vf_ICD1 _and Vf_ICD2 _kept at 2.5%, the other at 5%, of the original values. The plots of J_ICD1 _+ J_ICD2 _and J_ICL1 _+ J_ICL2 _against (I/K_I_) showed that I/K_I _ratios of about 477 (figure 3A) and 105 (figure 3B) respectively were required to reduce J_ICL1 _+ J_ICL2 _by 90%.

v=VfICL1ICITKM,ICIT−VrICL1SUCKM,SUCGLYKM,GLY(1+ICITKM,ICIT+SUCKM,SUC+GLYKM,GLY+ICITKM,ICITSUCKM,SUC+SUCKM,SUCGLYKM,GLY+IKI)     equation (1)
 MathType@MTEF@5@5@+=feaafiart1ev1aaatCvAUfKttLearuWrP9MDH5MBPbIqV92AaeXatLxBI9gBaebbnrfifHhDYfgasaacH8akY=wiFfYdH8Gipec8Eeeu0xXdbba9frFj0=OqFfea0dXdd9vqai=hGuQ8kuc9pgc9s8qqaq=dirpe0xb9q8qiLsFr0=vr0=vr0dc8meaabaqaciaacaGaaeqabaqabeGadaaakeaacqWG2bGDcqGH9aqpdaWcaaqaaiabdAfawjabdAgaMnaaBaaaleaacqWGjbqscqWGdbWqcqWGmbatcqaIXaqmaeqaaOWaaSaaaeaacqWGjbqscqWGdbWqcqWGjbqscqWGubavaeaacqWGlbWsdaWgaaWcbaGaemyta0KaeiilaWIaemysaKKaem4qamKaemysaKKaemivaqfabeaaaaGccqGHsislcqWGwbGvcqWGYbGCdaWgaaWcbaGaemysaKKaem4qamKaemitaWKaeGymaedabeaakmaalaaabaGaem4uamLaemyvauLaem4qameabaGaem4saS0aaSbaaSqaaiabd2eanjabcYcaSiabdofatjabdwfavjabdoeadbqabaaaaOWaaSaaaeaacqWGhbWrcqWGmbatcqWGzbqwaeaacqWGlbWsdaWgaaWcbaGaemyta0KaeiilaWIaem4raCKaemitaWKaemywaKfabeaaaaaakeaadaqadiabaeqabaGaeGymaeJaey4kaSYaaSaaaeaacqWGjbqscqWGdbWqcqWGjbqscqWGubavaeaacqWGlbWsdaWgaaWcbaGaemyta0KaeiilaWIaemysaKKaem4qamKaemysaKKaemivaqfabeaaaaGccqGHRaWkdaWcaaqaaiabdofatjabdwfavjabdoeadbqaaiabdUealnaaBaaaleaacqWGnbqtcqGGSaalcqWGtbWucqWGvbqvcqWGdbWqaeqaaaaakiabgUcaRmaalaaabaGaem4raCKaemitaWKaemywaKfabaGaem4saS0aaSbaaSqaaiabd2eanjabcYcaSiabdEeahjabdYeamjabdMfazbqabaaaaOGaey4kaScabaWaaSaaaeaacqWGjbqscqWGdbWqcqWGjbqscqWGubavaeaacqWGlbWsdaWgaaWcbaGaemyta0KaeiilaWIaemysaKKaem4qamKaemysaKKaemivaqfabeaaaaGcdaWcaaqaaiabdofatjabdwfavjabdoeadbqaaiabdUealnaaBaaaleaacqWGnbqtcqGGSaalcqWGtbWucqWGvbqvcqWGdbWqaeqaaaaakiabgUcaRmaalaaabaGaem4uamLaemyvauLaem4qameabaGaem4saS0aaSbaaSqaaiabd2eanjabcYcaSiabdofatjabdwfavjabdoeadbqabaaaaOWaaSaaaeaacqWGhbWrcqWGmbatcqWGzbqwaeaacqWGlbWsdaWgaaWcbaGaemyta0KaeiilaWIaem4raCKaemitaWKaemywaKfabeaaaaGccqGHRaWkdaWcaaqaaiabdMeajbqaaiabdUealnaaBaaaleaacqWGjbqsaeqaaaaaaaGccaGLOaGaayzkaaaaaiaaxMaacaWLjaGaeeyzauMaeeyCaeNaeeyDauNaeeyyaeMaeeiDaqNaeeyAaKMaee4Ba8MaeeOBa4MaeeiiaaYaaeWaceaacqaIXaqmaiaawIcacaGLPaaaaaa@C633@

An increase was observed in the efficiency of competitive inhibition of ICL1 and ICL2 with an increase in Vf_ICD1 _and Vf_ICD2 _from 2.5% to 5% of the original values, because at lower Vf_ICD1 _and Vf_ICD2_, inhibition of ICL1 and ICL2 leads to an increase in *iso*citrate concentration, nullifying the effect of competitive inhibition.

### Uncompetitive inhibition of ICLs

**Figure 4:**
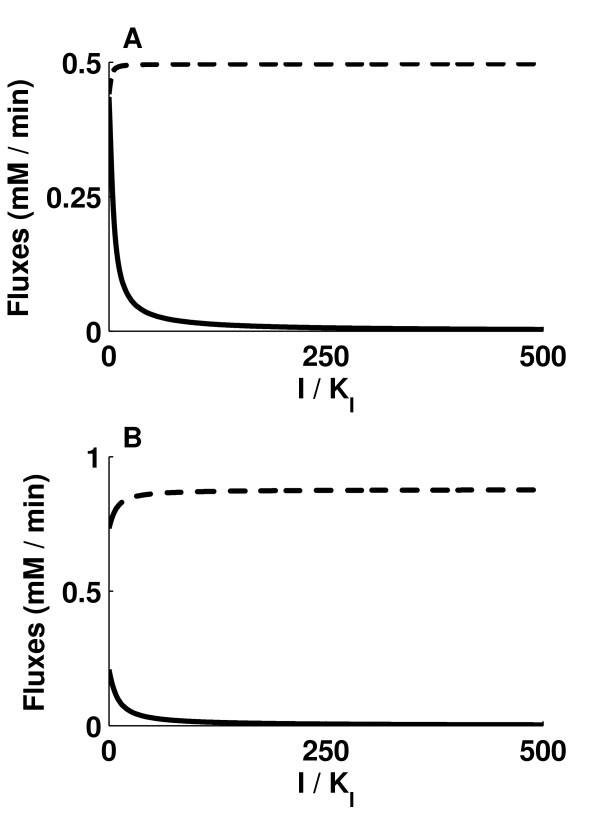
**Uncompetitive inhibition of ICLs by an inhibitor with concentration I and inhibitor constant K_I_**. Inhibition of ICL1 and ICL2, with Vf_ICD1 _and Vf_ICD2 _both kept at (A) 2.5% of the original values and (B) 5% of the original values. Broken line represents the sum of flux through ICD1 and ICD2, and solid line represents the sum of flux through ICL1 and ICL2. The effect of inhibitor is shown by varying the ratio of I/K_I_.

The rate equations of the ICL1 (equation (2)) and ICL2 reactions were modified to account for uncompetitive inhibition against *iso*citrate. The procedure used was similar to that described for competitive inhibition. The plots of J_ICD1 _+ J_ICD2 _and J_ICL1 _+ J_ICL2 _against (I/K_I_) showed that I/K_I _ratios of about 35 (figure 4A) and 71 (figure 4B) respectively were required to reduce J_ICL1 _+ J_ICL2 _by 90%. The corresponding reductions in J_ICL1 _+ J_ICL2 _by competitive inhibition of ICL1 and ICL2 were 52.4% and 86.2% respectively.

v=VfICL1ICITKM,ICIT−VrICL1SUCKM,SUCGLYKM,GLY(1+ICITKM,ICIT+ICITKM,ICITIKI+SUCKM,SUC+GLYKM,GLY+ICITKM,ICITSUCKM,SUC+SUCKM,SUCGLYKM,GLY)     equation (2)
 MathType@MTEF@5@5@+=feaafiart1ev1aaatCvAUfKttLearuWrP9MDH5MBPbIqV92AaeXatLxBI9gBaebbnrfifHhDYfgasaacH8akY=wiFfYdH8Gipec8Eeeu0xXdbba9frFj0=OqFfea0dXdd9vqai=hGuQ8kuc9pgc9s8qqaq=dirpe0xb9q8qiLsFr0=vr0=vr0dc8meaabaqaciaacaGaaeqabaqabeGadaaakeaacqWG2bGDcqGH9aqpdaWcaaqaaiabdAfawjabdAgaMnaaBaaaleaacqWGjbqscqWGdbWqcqWGmbatcqaIXaqmaeqaaOWaaSaaaeaacqWGjbqscqWGdbWqcqWGjbqscqWGubavaeaacqWGlbWsdaWgaaWcbaGaemyta0KaeiilaWIaemysaKKaem4qamKaemysaKKaemivaqfabeaaaaGccqGHsislcqWGwbGvcqWGYbGCdaWgaaWcbaGaemysaKKaem4qamKaemitaWKaeGymaedabeaakmaalaaabaGaem4uamLaemyvauLaem4qameabaGaem4saS0aaSbaaSqaaiabd2eanjabcYcaSiabdofatjabdwfavjabdoeadbqabaaaaOWaaSaaaeaacqWGhbWrcqWGmbatcqWGzbqwaeaacqWGlbWsdaWgaaWcbaGaemyta0KaeiilaWIaem4raCKaemitaWKaemywaKfabeaaaaaakeaadaqadiabaeqabaGaeGymaeJaey4kaSYaaSaaaeaacqWGjbqscqWGdbWqcqWGjbqscqWGubavaeaacqWGlbWsdaWgaaWcbaGaemyta0KaeiilaWIaemysaKKaem4qamKaemysaKKaemivaqfabeaaaaGccqGHRaWkdaWcaaqaaiabdMeajjabdoeadjabdMeajjabdsfaubqaaiabdUealnaaBaaaleaacqWGnbqtcqGGSaalcqWGjbqscqWGdbWqcqWGjbqscqWGubavaeqaaaaakmaalaaabaGaemysaKeabaGaem4saS0aaSbaaSqaaiabdMeajbqabaaaaOGaey4kaSYaaSaaaeaacqWGtbWucqWGvbqvcqWGdbWqaeaacqWGlbWsdaWgaaWcbaGaemyta0KaeiilaWIaem4uamLaemyvauLaem4qameabeaaaaGccqGHRaWkaeaadaWcaaqaaiabdEeahjabdYeamjabdMfazbqaaiabdUealnaaBaaaleaacqWGnbqtcqGGSaalcqWGhbWrcqWGmbatcqWGzbqwaeqaaaaakiabgUcaRmaalaaabaGaemysaKKaem4qamKaemysaKKaemivaqfabaGaem4saS0aaSbaaSqaaiabd2eanjabcYcaSiabdMeajjabdoeadjabdMeajjabdsfaubqabaaaaOWaaSaaaeaacqWGtbWucqWGvbqvcqWGdbWqaeaacqWGlbWsdaWgaaWcbaGaemyta0KaeiilaWIaem4uamLaemyvauLaem4qameabeaaaaGccqGHRaWkdaWcaaqaaiabdofatjabdwfavjabdoeadbqaaiabdUealnaaBaaaleaacqWGnbqtcqGGSaalcqWGtbWucqWGvbqvcqWGdbWqaeqaaaaakmaalaaabaGaem4raCKaemitaWKaemywaKfabaGaem4saS0aaSbaaSqaaiabd2eanjabcYcaSiabdEeahjabdYeamjabdMfazbqabaaaaaaakiaawIcacaGLPaaaaaGaaCzcaiaaxMaacqqGLbqzcqqGXbqCcqqG1bqDcqqGHbqycqqG0baDcqqGPbqAcqqGVbWBcqqGUbGBcqqGGaaidaqadiqaaiabikdaYaGaayjkaiaawMcaaaaa@D289@

In contrast to competitive inhibition of ICL1 and ICL2, the efficiency of uncompetitive inhibition decreased with an increase in Vf_ICD1 _and Vf_ICD2 _from 2.5% to 5% of the original values. This is because an increase in the Vmax of the ICDs leads to a decrease in *iso*citrate concentration, and hence to a decrease in the enzyme-substrate complex concentration. Because an uncompetitive inhibitor binds only to the enzyme-substrate complex, a decrease in enzyme-substrate complex concentration leads to a decrease in inhibitor binding, resulting in less inhibition.

The increase in efficiency of competitive inhibition with an increase in the Vmax of the ICDs leads to an alternative strategy for killing mycobacteria, i.e. by using a competitive inhibitor of ICL1 and ICL2 along with inhibition of ICD1-kinase and/or the proposed inactivator of ICD2. Inhibition of ICD1-kinase and/or proposed inactivator of ICD2 would increase the amount of active ICD1 and/or ICD2, i.e. would indirectly cause an increase in the Vmax of ICD1 and/or ICD2, thus indirectly improving the efficiency of competitive inhibition of the ICLs by the available *iso*citrate and reducing the competition between the substrate *iso*citrate and inhibitor. The points to note in this strategy are: (i) a competitive inhibitor of ICLs can serve the purpose; and (ii) the percentage inhibition of the ICD-kinase and/or proposed inactivator of ICD2 required here would be less than required to increase the amount of active ICD1 and/or ICD2 sufficiently to stop the flux through the glyoxylate bypass.

### Mixed inhibition of ICLs

**Figure 5:**
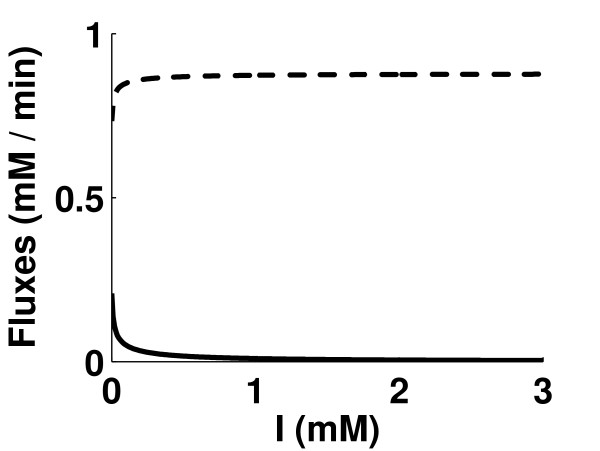
**Simulation of the effect of inhibition of both ICL1 and ICL2 by 3-nitropropionate (3-NP)**. Broken line represents the sum of flux through ICD1 and ICD2, and solid line represents the sum of flux through ICL1 and ICL2. Vf_ICD1 _and Vf_ICD2 _both kept at 5% of the original values during the simulation.

Here, an attempt has been made to simulate the inhibition of ICLs by 3-nitropropionate (3-NP), a dual-specific ICL inhibitor that is known to block the growth of mycobacteria in macrophages at a concentration of 0.1 mM [2]. 3-NP is competitive against succinate and uncompetitive against either glyoxylate or *iso*citrate [20]. The ICL1 and ICL2 rate equations were therefore modified to account for mixed inhibition (rate equation for ICL1 is shown in equation (3); 'I' denotes 3-NP concentration). A similar equation was used for ICL2. The inhibitor constants (K_I_) of 3-NP for ICL1 and ICL2 are 0.003 mM and 0.11 mM respectively [18]. Using these K_I _values, simulations were performed to study the effect of 3-NP concentration on J_ICD1 _+ J_ICD2 _and J_ICL1 _+ J_ICL2 _in the model (figure 5). Vf_ICD1 _and Vf_ICD2 _were kept at 5% of the original values during the simulation, driving the *iso*citrate towards the shunt (glyoxylate bypass) pathway. The results showed that a concentration of 0.38 mM 3-NP was required to reduce the *in vivo *flux through glyoxylate bypass by 90%. An almost 10-fold lower inhibitor concentration was required for 50% inhibition of ICL1 *in vitro *compared to the model (result not shown). A concentration of 0.1 mM, which experimentally blocks the growth of mycobacteria in macrophages [2], reduced the flux by 75.8%. It was also observed that a concentration of 3 mM was required to reduce the flux by 98.4%.

v=VfICL1ICITKM,ICIT−VrICL1SUCKM,SUCGLYKM,GLY(1+ICITKM,ICIT+ICITKM,ICITIKI+(SUCKM,SUC+IKI)+GLYKM,GLY+GLYKM,GLYIKI+ICITKM,ICITSUCKM,SUC+SUCKM,SUCGLYKM,GLY)     equation (3)
 MathType@MTEF@5@5@+=feaafiart1ev1aaatCvAUfKttLearuWrP9MDH5MBPbIqV92AaeXatLxBI9gBaebbnrfifHhDYfgasaacH8akY=wiFfYdH8Gipec8Eeeu0xXdbba9frFj0=OqFfea0dXdd9vqai=hGuQ8kuc9pgc9s8qqaq=dirpe0xb9q8qiLsFr0=vr0=vr0dc8meaabaqaciaacaGaaeqabaqabeGadaaakeaacqWG2bGDcqGH9aqpdaWcaaqaaiabdAfawjabdAgaMnaaBaaaleaacqWGjbqscqWGdbWqcqWGmbatcqaIXaqmaeqaaOWaaSaaaeaacqWGjbqscqWGdbWqcqWGjbqscqWGubavaeaacqWGlbWsdaWgaaWcbaGaemyta0KaeiilaWIaemysaKKaem4qamKaemysaKKaemivaqfabeaaaaGccqGHsislcqWGwbGvcqWGYbGCdaWgaaWcbaGaemysaKKaem4qamKaemitaWKaeGymaedabeaakmaalaaabaGaem4uamLaemyvauLaem4qameabaGaem4saS0aaSbaaSqaaiabd2eanjabcYcaSiabdofatjabdwfavjabdoeadbqabaaaaOWaaSaaaeaacqWGhbWrcqWGmbatcqWGzbqwaeaacqWGlbWsdaWgaaWcbaGaemyta0KaeiilaWIaem4raCKaemitaWKaemywaKfabeaaaaaakeaadaqadiabaeqabaGaeGymaeJaey4kaSYaaSaaaeaacqWGjbqscqWGdbWqcqWGjbqscqWGubavaeaacqWGlbWsdaWgaaWcbaGaemyta0KaeiilaWIaemysaKKaem4qamKaemysaKKaemivaqfabeaaaaGccqGHRaWkdaWcaaqaaiabdMeajjabdoeadjabdMeajjabdsfaubqaaiabdUealnaaBaaaleaacqWGnbqtcqGGSaalcqWGjbqscqWGdbWqcqWGjbqscqWGubavaeqaaaaakmaalaaabaGaemysaKeabaGaem4saS0aaSbaaSqaaiabdMeajbqabaaaaOGaey4kaSYaaeWaceaadaWcaaqaaiabdofatjabdwfavjabdoeadbqaaiabdUealnaaBaaaleaacqWGnbqtcqGGSaalcqWGtbWucqWGvbqvcqWGdbWqaeqaaaaakiabgUcaRmaalaaabaGaemysaKeabaGaem4saS0aaSbaaSqaaiabdMeajbqabaaaaaGccaGLOaGaayzkaaGaey4kaSYaaSaaaeaacqWGhbWrcqWGmbatcqWGzbqwaeaacqWGlbWsdaWgaaWcbaGaemyta0KaeiilaWIaem4raCKaemitaWKaemywaKfabeaaaaGccqGHRaWkaeaadaWcaaqaaiabdEeahjabdYeamjabdMfazbqaaiabdUealnaaBaaaleaacqWGnbqtcqGGSaalcqWGhbWrcqWGmbatcqWGzbqwaeqaaaaakmaalaaabaGaemysaKeabaGaem4saS0aaSbaaSqaaiabdMeajbqabaaaaOGaey4kaSYaaSaaaeaacqWGjbqscqWGdbWqcqWGjbqscqWGubavaeaacqWGlbWsdaWgaaWcbaGaemyta0KaeiilaWIaemysaKKaem4qamKaemysaKKaemivaqfabeaaaaGcdaWcaaqaaiabdofatjabdwfavjabdoeadbqaaiabdUealnaaBaaaleaacqWGnbqtcqGGSaalcqWGtbWucqWGvbqvcqWGdbWqaeqaaaaakiabgUcaRmaalaaabaGaem4uamLaemyvauLaem4qameabaGaem4saS0aaSbaaSqaaiabd2eanjabcYcaSiabdofatjabdwfavjabdoeadbqabaaaaOWaaSaaaeaacqWGhbWrcqWGmbatcqWGzbqwaeaacqWGlbWsdaWgaaWcbaGaemyta0KaeiilaWIaem4raCKaemitaWKaemywaKfabeaaaaaaaOGaayjkaiaawMcaaaaacaWLjaGaaCzcaiabbwgaLjabbghaXjabbwha1jabbggaHjabbsha0jabbMgaPjabb+gaVjabb6gaUjabbccaGmaabmGabaGaeG4mamdacaGLOaGaayzkaaaaaa@E75E@

Considering that we focused on the TCA cycle and glyoxylate bypass only, and that the model was built with a number of permissible assumptions, the results obtained agree satisfactorily with the experimental data. The observation that inhibition of ICLs results in no marked changes in the concentrations of any other metabolites in the model (result not shown), but to a decrease in the flux through glyoxylate bypass, indicates that the clearing of mycobacterial load from macrophages as observed by McKinney and co-workers [2] can be correlated with a decrease in the glyoxylate bypass flux, not with accumulation of any toxic metabolite.

## Conclusion

This study constitutes a proof of concept: one can use kinetic modeling of biochemical pathways to investigate potential drug targets and to infer the type of inhibition appropriate for eliminating the pathogen. The study highlights the difference between the inhibitor concentrations required *in vitro *and *in vivo *to inhibit the glyoxylate bypass pathway enzymes. The advantage of this approach to assessing drug targets is that it facilitates the study of systemic effect(s) of modulating the target enzyme(s) on the pathway. The applicability of the study is certainly limited by the approximations and assumptions made while constructing the models, but these should be overcome soon because the required data are accumulating rapidly in this post-genomic era.

## Methods

The steps in the construction of the kinetic model are described below.

### Biochemical reactions in the pathway

The biochemical reactions of the *E. coli *TCA cycle and glyoxylate bypass were obtained from EcoCyc [21], and those of *M. tuberculosis *from MetaCyc [22]. These reactions for the two organisms from the two different data sources were identical. A reaction branching from α-ketoglutarate (αKG = precursor; named SYN in the models) was added to both the *E. coli *and *M. tuberculosis *models to account for the fraction of αKG utilized for precursor biosynthesis (as shown by Zhao *et al*. [15] in *E. coli*). A set of two reactions catalyzed by α-ketoglutarate decarboxylase (KGD) and succinic semialdehyde dehydrogenase (SSADH) that together convert αKG to succinate (SUC) via succinic semialdehyde (SSA) was also included in the *M. tuberculosis *model. The model also accounted for the presence of two isoforms of ICD [17], ICD1 (Rv3339c) and ICD2 (Rv0066c), and two isoforms of ICL [17,18], ICL1 (Rv0467) and ICL2 (Rv1915 and Rv1916), in *M. tuberculosis *H37Rv strain. The requisite co-enzymes and co-factors were assumed to be present in large excess so their effects on the reaction rates in the models were ignored. The reactions considered in the construction of the models are shown in figure 1.

Recently, Nathan and co-workers failed to detect α-ketoglutarate dehydrogenase (KDH) activity in *M. tuberculosis *[13]. They suggested that Rv1248c, annotated as encoding SucA, the putative E1 component of KDH, encodes KGD and produces SSA. SSA is then converted by SSADH to SUC. This new finding was also incorporated into our study by constructing another model for *M. tuberculosis *(named *M. tuberculosis *model-2) in which the KDH reaction was removed (see figure 1).

### Reaction kinetics

Michaelis-Menten equations for one substrate and two-substrate reactions were used to describe the reaction kinetics in the models. The reversible Michaelis-Menten equation for two non-competing product-substrate couples is shown in equation (4) [23]:

v=VfS1KS1S2KS2−VrP1KP1P2KP2(1+S1KS1+P1KP1)(1+S2KS2+P2KP2)     equation (4)
 MathType@MTEF@5@5@+=feaafiart1ev1aaatCvAUfKttLearuWrP9MDH5MBPbIqV92AaeXatLxBI9gBaebbnrfifHhDYfgasaacH8akY=wiFfYdH8Gipec8Eeeu0xXdbba9frFj0=OqFfea0dXdd9vqai=hGuQ8kuc9pgc9s8qqaq=dirpe0xb9q8qiLsFr0=vr0=vr0dc8meaabaqaciaacaGaaeqabaqabeGadaaakeaacqWG2bGDcqGH9aqpdaWcaaqaaiabdAfawjabdAgaMnaalaaabaGaem4uam1aaSbaaSqaaiabigdaXaqabaaakeaacqWGlbWsdaWgaaWcbaGaem4uam1aaSbaaWqaaiabigdaXaqabaaaleqaaaaakmaalaaabaGaem4uam1aaSbaaSqaaiabikdaYaqabaaakeaacqWGlbWsdaWgaaWcbaGaem4uam1aaSbaaWqaaiabikdaYaqabaaaleqaaaaakiabgkHiTiabdAfawjabdkhaYnaalaaabaGaemiuaa1aaSbaaSqaaiabigdaXaqabaaakeaacqWGlbWsdaWgaaWcbaGaemiuaa1aaSbaaWqaaiabigdaXaqabaaaleqaaaaakmaalaaabaGaemiuaa1aaSbaaSqaaiabikdaYaqabaaakeaacqWGlbWsdaWgaaWcbaGaemiuaa1aaSbaaWqaaiabikdaYaqabaaaleqaaaaaaOqaamaabmGabaGaeGymaeJaey4kaSYaaSaaaeaacqWGtbWudaWgaaWcbaGaeGymaedabeaaaOqaaiabdUealnaaBaaaleaacqWGtbWudaWgaaadbaGaeGymaedabeaaaSqabaaaaOGaey4kaSYaaSaaaeaacqWGqbaudaWgaaWcbaGaeGymaedabeaaaOqaaiabdUealnaaBaaaleaacqWGqbaudaWgaaadbaGaeGymaedabeaaaSqabaaaaaGccaGLOaGaayzkaaWaaeWaceaacqaIXaqmcqGHRaWkdaWcaaqaaiabdofatnaaBaaaleaacqaIYaGmaeqaaaGcbaGaem4saS0aaSbaaSqaaiabdofatnaaBaaameaacqaIYaGmaeqaaaWcbeaaaaGccqGHRaWkdaWcaaqaaiabdcfaqnaaBaaaleaacqaIYaGmaeqaaaGcbaGaem4saS0aaSbaaSqaaiabdcfaqnaaBaaameaacqaIYaGmaeqaaaWcbeaaaaaakiaawIcacaGLPaaaaaGaaCzcaiaaxMaacqqGLbqzcqqGXbqCcqqG1bqDcqqGHbqycqqG0baDcqqGPbqAcqqGVbWBcqqGUbGBcqqGGaaidaqadiqaaiabisda0aGaayjkaiaawMcaaaaa@7DC6@

where v = net rate of the reaction; Vf, Vr = maximal rates of the forward and reverse reaction, respectively; S_1_, S_2 _= concentrations of substrates S_1 _and S_2 _respectively; P_1_, P_2 _= concentrations of products P_1 _and P_2 _respectively; K_S1_, K_S2_, K_P1_, K_P2 _= Michaelis-Menten constants for S_1_, S_2_, P_1 _and P_2 _respectively.

The only reaction in which a different kinetic equation was used was the reaction: ICIT = SUC + glyoxylate (GLY), catalyzed by ICL. This is known to occur by an ordered uni-bi mechanism [24] as described by Bakker et. al [7].

### Parameters of the models

The kinetic parameters of the enzymes in the models (see [additional file 1: Kinetic constants of the enzymes in *E. coli *model'] and [additional file 2: Kinetic constants of the enzymes in *M. tuberculosis *model-1 and model-2]) were either obtained from publicly available databases, namely CyberCell Database (CCDB) [25] and BRENDA [26], or extracted from the literature. The maximal reaction rates (Vmax) expressed in nmol/min/mg protein were converted to mM/min by taking the intracellular volume of a bacterial cell as 2 × 10^-12 ^ml [27] and the total protein content as 3.2 × 10^-10 ^mg [28]. We were interested in studying the reactions of the pathway in the catabolic direction, i.e. the direction in which it usually works in the cell; so in cases where the value of Vr was not available it was taken as a fraction of Vf (after some trial and error, Vr = Vf/100). In cases where reverse reaction had been monitored and Vr reported, Vf was taken as equal to Vr. Where a K_M _was not available, usually for a reverse reaction, it was assumed to be equal to 10 × K_M _of the substrate from which that product was formed (by the same logic as used for the Vr values). The metabolites acetyl-CoA, oxaloacetate and CoA were considered as boundary metabolites, so their concentrations were fixed in the simulations. The initial concentration of each variable metabolite was taken as 2 × K_M _for the reaction for which that metabolite is a substrate (except for those metabolites of which the concentrations were known; see table 1).

In the *E. coli *model, the carbon flux through the pathway was predicted under two growth conditions, viz. growth on glucose and acetate as carbon sources. Most enzyme kinetic parameters are available for *E. coli *grown on glucose, but it is also necessary to estimate the enzyme kinetic parameters for the acetate condition. The changes in *E. coli *gene expression when growth shifts from glucose to acetate were described by Oh *et al*. [14]. Assuming that the change in mRNA level leads to a proportional change in protein level (enzyme level in our study), there would be a proportional change in the Vmax of that enzyme (because Vmax is proportional to the amount of enzyme). Thus, using the Vmax values of enzymes under the glucose condition and the fold change in gene expression of the corresponding enzymes, the Vmax values under the acetate condition were calculated.

### Calculation of Vmax from gene expression data

Let, the expression levels of a gene g1 under the acetate and glucose conditions be g1_a _and g1_g _respectively. Therefore, the fold change when growth shifts from glucose to acetate is n = g1_a_/g1_g_. Taking account of the assumption that a change in mRNA level leads to a proportional change in protein level,

p1_a_/p1_g _= g1_a_/g1_g _= n     equation (5)

where p1 is the amount of the protein encoded by g1 and the subscripts 'a' and 'g' denote its level in acetate and glucose respectively

Since Vmax = kcat × E (where kcat = turnover number, E = amount of enzyme catalyzing the reaction) and kcat is a constant, Vmax α E

Therefore, from equation (5), Vmax_a_/Vmax_g _= n

(where Vmax_a_, Vmax_g _= Vmax of the enzyme in acetate and glucose respectively)

or Vmax_a _= n × Vmax_g_

Thus, using the values of n and Vmax_g_, Vmax_a _values were calculated and used as parameters for the model to simulate the condition of growth on acetate as the carbon source.

The rate of the SYN reaction was maintained at 0.188 times (for glucose condition) and 0.0341 times (for acetate condition) the rate of the ICD reaction in the *E. coli *model, as shown experimentally [15]. Owing to the unavailability of data for *M. tuberculosis*, the rate of the SYN reaction was maintained at that under acetate conditions in *E. coli*. The kinetic parameters for *M. tuberculosis *KDH were also assumed to be same as for *E. coli*. As ICL activity in persistent mycobacteria is 4 times that in the normal condition [28], the concentration of the ICLs were taken as 4 times those in normal conditions.

### Computation

Simulations were performed by writing scripts for Jarnac 2.14 [29]. First, steady states were calculated, then – starting from the steady state solution for each model – a time-dependent simulation was performed to test the stability of the steady state. We checked that the program Gepasi 3.30 [30] generates the same results as Jarnac given the same input, but we continued our work with Jarnac because it offered us the flexibility of writing our own scripts.

The fluxes computed from the models were expressed in mM/min. To compare the steady state fluxes of the *E. coli *model with experimental findings [15], they were converted to the units in which experimental fluxes were expressed. The experimental fluxes were expressed relative to (a) molar glucose uptake or (b) molar acetate uptake rate depending on the carbon source. The following steps were used to convert the units: flux through citrate synthase during growth on glucose = 50; flux through citrate synthase during growth on glucose in the model = 4.187 mM/min; hence, conversion factor *x *= (50)/(4.187 mM/min). Using this conversion factor (*x*), all the fluxes computed from the model were converted to the units in which experimental fluxes were expressed.

Example: flux through α-ketoglutarate dehydrogenase (KDH) reaction step in the model = 3.394 mM/min = (3.394 mM/min) × (*x *min/mM) = 40.5.

A similar conversion factor was calculated for growth on acetate using flux through the citrate synthase step.

## Abbreviations

ICL, *iso*citrate lyase; ACN, aconitase; αKG, α-ketoglutarate; CS, citrate synthase; FUM, fumarase; GLY, glyoxylate; I, inhibitor concentration; ICD, *iso*citrate dehydrogenase; ICIT, *iso*citrate; J_ICD1_, flux through ICD1; J_ICD2_, flux through ICD2; J_ICL1_, flux through ICL1; J_ICL2_, flux through ICL2; KDH, α-ketoglutarate dehydrogenase; KGD, α-ketoglutarate decarboxylase; K_I_, inhibitor constant of inhibitor I; MCA, Metabolic Control Analysis; MDH, malate dehydrogenase; MS, malate synthase; 3-NP, 3-nitropropionate; ScAS, succinyl-CoA synthetase; SDH, succinate dehydrogenase; SSA, succinic semialdehyde; SSADH, succinic semialdehyde dehydrogenase; SUC, succinate; TCA, tricarboxylic acid; Vf, maximal rate of the forward reaction; Vf_ICD1_, Vmax of the reaction catalyzed by ICD1 in the forward direction; Vf_ICD2_, Vmax of the reaction catalyzed by ICD2 in the forward direction; Vmax, maximal rate of an enzymatic reaction; Vr, maximal rate of the reverse reaction.

## Competing interests

The author(s) declare that they have no competing interests.

## Authors' contributions

VKS has contributed in developing the models, analysis and interpretation of data, and writing the manuscript. IG was involved in the overall design of this study, critical analysis and interpretation of the data, and revision of the draft of the manuscript.

## Supplementary Material

Additional File 1 **Kinetic constants of the enzymes in *E. coli *model**. Additional file 1 contains a table that enlist the kinetic constants of the enzymes in *E. coli *model. Click here for file

Additional File 2 **Kinetic constants of the enzymes in *M. tuberculosis *model-1 and model-2**. Additional file 2 contains a table that enlist kinetic constants of the enzymes in *M. tuberculosis *models. Click here for file
