# Supplementary material for: Kinetic modeling of tricarboxylic acid cycle and glyoxylate bypass in Mycobacterium tuberculosis, and its application to assessment of drug targets
Source: Theor Biol Med Model. 2006 Aug 3;3:27. doi: 10.1186/1742-4682-3-27 (PMC1563452; doi:10.1186/1742-4682-3-27)
Supplement: Additional File 1 — Kinetic constants of the enzymes in E. coli model. Additional file 1 contains a table that enlist the kinetic constants of the enzymes in E. coli model. [file 1742-4682-3-27-S1.pdf]

Additional Table 1 - Kinetic constants of the enzymes in *E. coli* model. Vmax refers to maximal rate of an enzymatic reaction. K_{S1} and K_{S2} refer to the K_M values of substrates, and, K_{P1} and K_{P2} refer to the K_M values of products of the reactions. They are numbered in the order they appear in the chemical equations shown in the table.

Reaction	Enzyme	Abbreviation	Vmax (in nmol/min/mg protein)	Vmax in glucose condition (mM/min)	Ratio of mRNA levels [1] in acetate and glucose condition	Vmax in acetate condition (mM/min)	K_{S1} (mM)	K_{S2} (mM)	K_{P1} (mM)	K_{P2} (mM)
ACA + OAA = COA + CIT	citrate synthase	CS	570 [2]	91.2	4.9	446.88	0.03 [3]	0.07 [3]	0.3	0.7
CIT = ICIT	aconitase	ACN	-	91.2[a]	acnA=1.5, acnB=6.9[b]	629.28	1.7[c] [4]		3.33 [c] [4]	
ICIT = aKG	*iso*citrate dehydrogenase	ICD	92 [2]	14.72	1.8	6.625[d]	0.008 [5]		0.13[e] [4]	
aKG = SCA	α-ketoglutarate dehydrogenase complex	KDH	224 [2]	35.84	1.6[f]	57.344	0.1 [4]		1	
SCA = SUC	succinyl-CoA synthetase	ScAS	20 [2]	3.5[g]	2.8[f]	8.96	0.02[h] [4]		5[i] [4]	
SUC = FA	succinate dehydrogenase	SDH	46.1 [2]	7.38	2.4[j]	17.7	0.02 [3]		0.4 [3]	
FA = MAL	fumarase	FUM	279 [2]	44.64	3.5	156.24	0.15 [4]		0.04 [4]	
MAL = OAA	malate dehydrogenase	MDH	2229 [2]	356.64	3.9	1390.9	2.6 [4]		0.04 [4]	
ICIT = SUC + GLY	*iso*citrate lyase	ICL[k]	-	1.9[l]	15	28.5	0.604 [5]		0.59 [4]	0.13 [4]
GLY + ACA = MAL + COA	malate synthase	MS[m]	-	1.9	17	28.5	2 [3]	0.01 [3]	1 [4]	0.1

Notes for additional table 1

[a]Value assumed to be equal to that of CS
[b]Since acnB is the main catalytic enzyme [6], acnB values was used
[c]Value taken from *Bacillus subtilis*
[d]Multiply the resultant (Vmax x fold change) by ¼ because only 1/4th ICD is active under growth on acetate [7], so value=6.625 mM/min
[e]Value taken from *Sus scrofa*
[f]Since the subunit of enzyme which is in lowest concentration will control the number of functional enzyme molecules, the subunit which has the lowest fold change was used for the calculation of Vmax of the enzyme under growth on acetate
[g]Minor adjustment was required, so 3.5 mM/min was used instead of 3.2 mM/min

[h]Value taken from *Calliphoridae*
[i]Value taken from *Nitrosomonas europaea*
[j]2.4 used for calculation because this is the fold change in the gene coding for catalytic subunit of the SDH [6]
[k]Calculation of Vmax of ICL: ICL concentration = 0.2 mM (when grown on acetate) [5]; Turnover number = 1710 min (*E. coli* ML308 [8])

So, Vmax = 1710 x (0.2 / (4 (since ICL is a tetramer) x 3 (since ICD intracellular concentration reported by Walsh et. al [5] is 3 times higher than that calculated from protein copy number [3], so it was assumed that there would be similar difference in ICL concentration reported by Walsh et. al [5], and thus, it was divided by 3)). So, Vmax = 1710 x 0.2 / 12 = 28.5 mM/min

[l]Calculated from Vmax of ICL in acetate condition, by dividing Vmax by fold change in the gene expression when growth shifts from glucose to acetate [1]
[m]Vmax of MS was taken equal to Vmax of ICL because the genes coding for MS and ICL belong to the same operon (operon: *aceBAK*)

References

1. Oh MK, Rohlin L, Kao KC, Liao JC: **Global expression profiling of acetate-grown *Escherichia coli*.** *J Biol Chem* 2002, 277: 13175-13183
2. Tian J, Bryk R, Itoh M, Suematsu M, Nathan C: **Variant tricarboxylic acid cycle in *Mycobacterium tuberculosis*: Identification of α-ketoglutarate decarboxylase.** *Proc Natl Acad Sci U S A* 2005, 102: 10670-10675
3. Sundararaj S, Guo A, Habibi-Nazhad B, Rouani M, Stothard P, Ellison M, Wishart DS: **The CyberCell Database (CCDB): a comprehensive, self-updating, relational database to coordinate and facilitate *in silico* modeling of *Escherichia coli*.** *Nucleic Acids Res* 2004, 32: D293-D295
4. Schomburg I, Chang A, Schomburg D: **BRENDA, enzyme data and metabolic information.** *Nucleic Acids Res* 2002, 30: 47-49
5. Walsh K, Koshland DE Jr: **Determination of flux through the branch point of two metabolic cycles the tricarboxylic acid cycle and the glyoxylate shunt.** *J Biol Chem* 1984, 259: 9646-9654
6. Keseler IM, Collado-Vides J, Gama-Castro S, Ingraham J, Paley S, Paulsen IT, Peralta-Gil M, Karp PD: **EcoCyc: a comprehensive database resource for *Escherichia coli*.** *Nucleic Acids Res* 2005, 33: D334-D337
7. LaPorte DC, Walsh K, Koshland DE Jr: **The branch point effect ultrasensitivity and subsensitivity to metabolic control.** *J Biol Chem* 1984, 259: 14068-14075
8. Robertson AG, Nimmo HG: **Site-directed mutagenesis of cysteine-195 in isocitrate lyase from *Escherichia coli* ML308.** *Biochem J* 1995, 305: 239-244

Abbreviations of metabolites

ACA	acetyl-CoA
OAA	oxaloacetate
COA	CoA
CIT	citrate
ICIT	isocitrate
aKG	α-ketoglutarate
SCA	succinyl-CoA
SUC	succinate
FA	fumarate
MAL	malate
GLY	glyoxylate
