# Supplementary material for: Kinetic modeling of tricarboxylic acid cycle and glyoxylate bypass in Mycobacterium tuberculosis, and its application to assessment of drug targets
Source: Theor Biol Med Model. 2006 Aug 3;3:27. doi: 10.1186/1742-4682-3-27 (PMC1563452; doi:10.1186/1742-4682-3-27)
Supplement: Additional File 2 — Kinetic constants of the enzymes in M. tuberculosis model-1 and model-2. Additional file 2 contains a table that enlist kinetic constants of the enzymes in M. tuberculosis models. [file 1742-4682-3-27-S2.pdf]

Additional Table 2 - Kinetic constants of the enzymes in *M. tuberculosis* model-1 and model-2. Vmax refers to maximal rate of an enzymatic reaction. K_{S1} and K_{S2} refer to the K_M values of substrates, and, K_{P1} and K_{P2} refer to the K_M values of products of the reactions. They are numbered in the order they appear in the chemical equations shown in the table.

Reaction	Enzyme	Abbreviation	Vmax (in nmol/min/mg protein)	Vmax (mM/min)	K_{S1} (mM)	K_{S2} (mM)	K_{P1} (mM)	K_{P2} (mM)
ACA + OAA = COA + CIT	citrate synthase	CS	405 [1]	64.8	0.05 [2]	0.012[a] [2]	0.5	0.12
CIT = ICIT	aconitase	ACN	195 [1]	31.2	1.7[b] [2]		0.7[c] [2]	
ICIT = aKG	*iso*citrate dehydrogenase 1	ICD1	64 [1]	10.2	0.03 [2]		0.3	
ICIT = aKG	*iso*citrate dehydrogenase 2	ICD2	-	9.965[d]	0.06[d]		0.6	
aKG = SCA[e]	α-ketoglutarate dehydrogenase complex	KDH	-	57.344[f]	0.1[f]		1	
SCA = SUC	succinyl-CoA synthetase	ScAS	7.5 [1]	1.2	0.02[g] [2]		5[h] [2]	
aKG = SSA	α-ketoglutarate decarboxylase	KGD	-	48.3[i]	0.48 [1]		4.8	
SSA = SUC	succinic semialdehyde dehydrogenase	SSADH	40.7 [1]	6.51	0.015[j] [2]		0.15	
SUC = FA	succinate dehydrogenase	SDH	6.4 [1]	1.02	0.15[k] [2]		0.12[k] [2]	
FA = MAL	fumarase	FUM	548 [1]	87.7	0.25[l] [2]		2.38[l] [2]	
MAL = OAA	malate dehydrogenase	MDH	1149 [1]	184	0.833[k] [2]		0.0443[k] [2]	
ICIT = SUC + GLY	*iso*citrate lyase 1	ICL1	1.83 [3]	1.172[m]	0.145 [2]		0.59[f]	0.13[f]
ICIT = SUC + GLY	*iso*citrate lyase 2	ICL2	-	0.391[n]	1.3 [4]		5.9[o]	1.3[o]
GLY + ACA = MAL + COA	malate synthase	MS	125 [3]	20	0.057 [2]	0.03 [2]	1[f]	0.1[f]

Notes for additional table 2

[a]Value taken from *Bacillus megaterium*
[b]Value taken from *Bacillus subtilis*
[c]Value taken from *Bacillus cereus*

[d]Banerjee et. al [5] estimated the Vmax of purified ICD1 and ICD2, and Tian et. al [1] estimated the Vmax of ICD1 in cell lysate. Using the ratio of Vmax of purified ICD2 to Vmax of purified ICD1, and the Vmax of ICD1 in cell lysate, the Vmax of ICD2 in cell lysate was estimated. Similar logic was used to estimate the K_M value of ICIT for ICD2

[e]This reaction is present only in *M. tuberculosis* model-1

[f]Value taken from *E. coli*

[g]Value taken from *Calliphoridae*

[h]Value taken from *Nitrosomonas europaea*

[i]Value was not available in cell lysate condition, so average of the Vmax of all TCA cycle enzymes was used

[j]Value taken from *Sus scrofa*

[k]Value taken from *Mycobacterium phlei*

[l]Value taken from bacterium

[m]Multiply the calculated Vmax (=0.293 mM/min) by 4 because ICL activity increases 4-fold in persistent mycobacteria [3]

[n]Logic similar to that used for the estimation of Vmax for ICD2 was used to estimate Vmax for ICL2 and K_M value of ICIT for ICL2. The data on purified ICL1 and ICL2 were obtained from Bentrup et. al [4])

[o]Since the K_M of ICIT for ICL2 is 10 times the K_M of ICIT for ICL1 [4], the K_M of the products i.e. SUC and GLY were also increased 10 fold.

References

1. Tian J, Bryk R, Itoh M, Suematsu M, Nathan C: **Variant tricarboxylic acid cycle in *Mycobacterium tuberculosis*: Identification of α-ketoglutarate decarboxylase.** *Proc Natl Acad Sci U S A* 2005, 102: 10670-10675
2. Schomburg I, Chang A, Schomburg D: **BRENDA, enzyme data and metabolic information.** *Nucleic Acids Res* 2002, 30: 47-49
3. Wayne LG, Lin KY: **Glyoxylate metabolism and adaptation of *Mycobacterium tuberculosis* to survival under anaerobic conditions.** *Infect Immun* 1982, 37: 1042-1049
4. Höner zu Bentrup K, Miczak A, Swenson DL, Russell DG: **Characterization of activity and expression of isocitrate lyase in *Mycobacterium avium* and *Mycobacterium tuberculosis*.** *J Bacteriol* 1999, 181: 7161-7167
5. Banerjee S, Nandyala A, Podili, R, Katoch VM, Hasnain SE: **Comparison of *Mycobacterium tuberculosis* isocitrate dehydrogenases (ICD-1 and ICD-2) reveals differences in coenzyme affinity, oligomeric state, pH tolerance and phylogenetic affiliation.** *BMC Biochem* 2005, 6: 20

Abbreviations of metabolites

ACA	acetyl-CoA
OAA	oxaloacetate
COA	CoA
CIT	citrate
ICIT	isocitrate
aKG	α-ketoglutarate
SCA	succinyl-CoA
SUC	succinate
FA	fumarate
MAL	malate
GLY	glyoxylate
SSA	succinic semialdehyde
